# Scientific requisites for academic advancements in Italy: time to change the rules

**DOI:** 10.1007/s13304-023-01612-6

**Published:** 2023-08-03

**Authors:** D. F. Altomare, G. Galizia, A. Mingoli, M. Raffaelli, F. Roviello

**Affiliations:** 1 National Scientific Habilitation Committee 2021-2023, Bari, Italy; 2 Surgical Unit “M.Rubino” DiMePRE-J, University of Bari, Bari, Italy; 3 Division of Gastrointestinal Tract Oncological Surgery, University of Naples ‘Luigi Vanvitelli’, Naples, Italy; 4 Department of Surgery, Sapienza University, Policlinico Umberto I, Rome, Italy; 5 U.O. Chirurgia Endocrina e Metabolica, Fondazione Policlinico Universitario Agostino Gemelli IRCCS, Rome, Italy; 6 Unit of General Surgery and Surgical Oncology, Department of Medicine, Surgery and Neurosciences, University of Siena, Siena, Italy

**Keywords:** Scientific habilitation, H index, Authorship

Progression of one’s academic career in Italy is regulated by the recent law n. 240 of 2010, which states that candidates must first obtain the national scientific habilitation (NSH) in order to be able to become associate or full professor. The selection of the eligible candidates is performed by a commission consisting of 5 full professors of the discipline of interest for a 2-year period, even if this limit could be extended without the agreement of the commission.

Admission of candidates to the national competition for the NSH is conditioned by the possession of 3 bibliometric indexes, namely a minimum number of papers published, a minimum number of citations and H index in a time frame of 5 or 10 years depending of the position required.

Candidates admitted are then evaluated on the basis of the achievements of at least 3 requirements previously selected by the commission and on the basis of their scientific activity, while the analysis of the quality of the scientific activity is based on the assessment of the best 12 or 16 papers that better represent the authors’ contribution to scientific knowledge.

In an effort to standardize the evaluation of candidates from different disciplines (namely scientific and disciplinary branches—SSD), the legislator has identified ten qualifications and has established that each Commission must select at least six of them. Therefore, the Commission has the power to select a variable number of titles designating more restrictive or more inclusive criteria.

It is immediately evident that candidates belonging to different SSD cannot be evaluated using the same titles. Instead, it would be appropriate to identify, for each SSD, the qualifications that best characterize the requisites necessary to become professor in that discipline. Furthermore, two successive Commissions could potentially select different titles, and therefore, the same candidate could be evaluated differently by the two commissions.

This is quite different from some Nord Europe countries such as Great Britain, Germany or Sweden, where new professors are appointed directly by the Universities on the base of their scientific curriculum (relevant paper in high Impact factor journals, important grant of research, new patents and so on), while in Spain (https://www.aneca.es/) and France, the procedures are similar to the Italians although with different rules.

However, several weakness points in these rules could contribute to the risk of production of unmerited positive judgements allowing inadequate candidates to access the position of associate or full professors, even if the nomination of new professors in Italy is finally achieved after a regular competition called for by single Universities.

A first consideration is that the basic requisite that candidates must have got the Medical Doctor Degree and the Diploma of Specialist in General Surgery is missing from the rules, and candidates who, from the beginning have built their career waiting several years, working in precarious conditions such as lecturers, research fellows, and assistant professors with fixed-term employment and have been trained to do research and teach to students, are not favored in this competition compared to doctors who dedicated their work exclusively to the patient’s assistance in hospitals.

On the other hand, for the medical specialties involving surgery, the surgical skillness and workload should also be considered, since for these disciplines, teaching, research, and clinical practice are so closely related to each other, that it is not conceivable to teach surgery without having great surgical experience.

However, the main drawbacks are related to the 3 bibliometric indexes.

The crossbar of the 3 bibliometric indexes requested to be admitted to the national scientific evaluation are too easily achieved, considering the increasing number of papers continuously published, new journals (online, open, etc.) available, and the active work of the “citations farms”.

Furthermore, all these indexes can be heavily affected by bias, misconducts, and even true frauds in research which is not just an Italian problem but involve all the Occidentalized countries.

The large and commonly accepted (mal)practice of gift authorship, which is tolerated even by very good researchers and felt as a venial sin, is responsible for an enormous number of unmerited attributions of papers to people who had nothing to do with the study or have not taken part sufficiently in it, thus not meeting the criteria for authorship claimed by the majority of the scientific journals [1]. This research misconduct in the field of Surgery has recently been pointed out by several studies [2, 3].

Solution to this bias is difficult because it involves the honesty of any researcher and their awareness of the potential dangerousness of this practice, wrongly considered a victimless behavior. However, the decision taken a few years ago by some journals to limit the number of authors to 6 or 8 unless clearly motivated was a shareable attempt to fix the problem, but it has inexplicably been virtually abandoned.

The pervasive phenomenon of the “big data papers” has recently introduced a new and destructive pickaxe to the concept of authorship. While the advantages of getting an enormous amount of data in a short period of time are clear and undeniable, the counterpart to convince hundreds or thousands of doctors to share their data is the promise to recognize their contribution with a gift authorship. In the last decade, favored by the Covid pandemic, a remarkable number of “big data” papers have been published in high reputation journals, sometimes giving important contribution to understanding the effects of the COVID 19 pandemic and its management [4]. As a consequence, each “author” obtained a rapid increase in the number of papers published in journals with high impact factor, which had a great chance to be quoted by thousands of other authors, and, consequently, increasing the H index.

The authorship should be limited to those who really meet the criteria for it, namely those who contributed by conceiving and designing the study, writing the paper, analysis of the data, intellectual contribution to the analysis and discussion of the data, final revision and approval of the final draft. A possible solution to the problem could be the exclusion from Scopus or Web of Science those (collaborators, etc.) who do not meet the authorship criteria, such as collaborators.

Actually, the criteria for authorship above mentioned still result inadequate when the academic role involves the category of surgeons. In fact, a new professional figure of author has recently been outlined: the medical writer. This is usually a medical doctor expert in the management of database and statistical elaboration of the data and methodology of research, but without any personal experience as a surgeon. These people offer their expertise in exchange for the authorship of the paper aiming to become professor of surgery. This is deeply different from some agencies present on the marked who offers critical and linguistic revision of scientific paper, since their work is compensated with money as a normal working activity (Fig. 1).

**Fig. 1:**
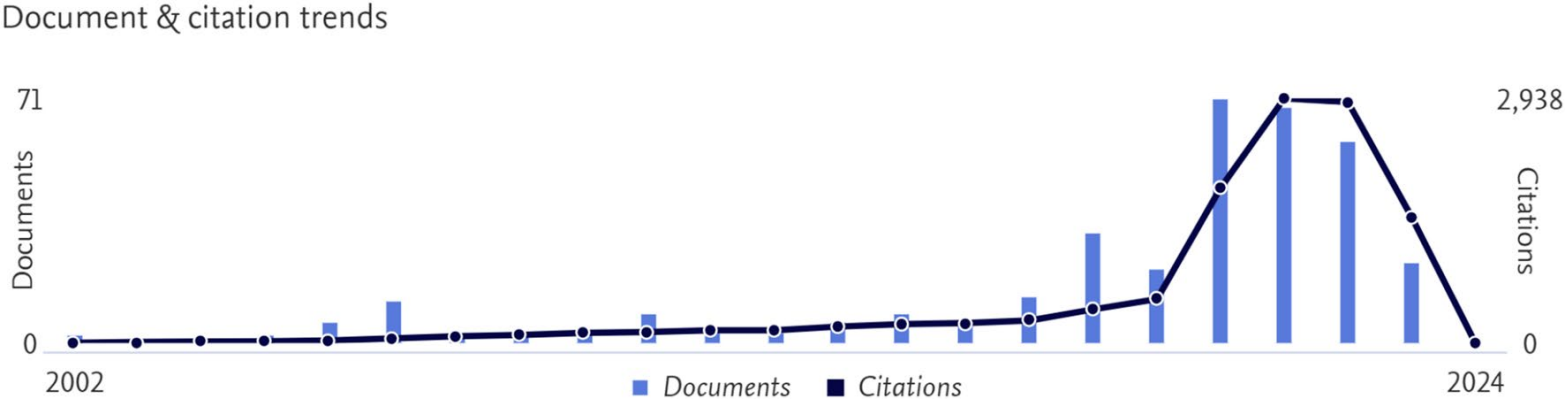
Typical time-trend of the number of publication during the COVID 19 pandemic. After about 20 years with a mediocre number of publications produced per year, suddenly during the COVID 19 pandemic there are about 70 papers per year, one every 5 days

A further threat in medical writing could come from the potential misuse of the AI to help generate content, analyze data, write discussion, edit the manuscript and so on). For example, using ChatGPT it could be possible to set up a review paper in a few hours by selecting the right questions. In an attempt to limit this incoming problem some Journals (i.e., Updates in Surgery) recommend that “The use of AI must be disclosed both in cover letters to editors and in the Methods or Acknowledgements”.

This controversial issue has recently been focused by Bonora [5] who stated that “the scientific progress is founded on original experiments and original papers”. “Analysts and “novelists” are fashionable, but they could not exist without “scientists”.

The second bibliometric index such as the number of citations in the time frame considered can also be affected by factors unrelated to the quality of the research.

Good papers published within 1–2 years have poor probability of being quoted by other authors representing an unfair penalty, especially for young researchers. On the other hand, the common practice of the reciprocal citation and the self-citation strictly related with the diffusion of big data papers with thousands of authors represents another plague for the correct evaluation of candidates.

To fix this problem, self-citations must be calculated and excluded from Scopus in the assessment of the H index. However, this is particularly difficult, or even impossible, if the authors participated to the “big-data papers” discussed above. Indeed, when the co-authors are thousands, Scopus platform is unable to exclude self-citations.

Another important issue is the circle citation or farm citation, also known as citation cartels which consists of authors who routinely and massively self-cite or cite each other in order to increase the impact of their publications. This unethical behavior should be punished when demonstrated.

Furthermore, some studies such as reviews or meta-analyses, surveys, position statements and guidelines have a very high chance of being quoted, because they summarize the most recent data of any topic. However, since they do not truly represent an original scientific contribution, rather an analysis of the pertinent original publications, they should be excluded in the calculation of the citations or adequately weighted, or, better, underweighted, in the analysis of any single candidate bibliometric indexes.

The third bibliometric index is the Hirsch Index (H index) which has become largely adopted in the last decades as the best indicator to assess an author’s performance. However, it suffers of several limitations. In fact, it can have different values according to the citation database used (Scopus/Web of Sciences), and negative or criticized papers are also considered, contributing to its increase.

Calculation of the H index cannot ignore the frequent “gift authorship” and the “Matthew effect” [6], neither consider the number of authors/paper which can explain the large differences in h-index between disciplines (in fact the authors from life science tend to publish more and shorter articles, including larger number of co-authors than the authors in the Social sciences), and for that reason the D (discipline) index was proposed and adopted.

Finally, the H index does not consider the length of academic life, which clearly favors oldest authors.

To overcome these limitations, two modified H indexes have been recently proposed, the hI,norm and the hI,annual. The hI,norm [7] is an individual h-index calculated by normalizing the number of citations for each paper by dividing the number of citations by the number of authors for that paper,. The hI,annual (or hIa) addresses the problem of comparing academics with different career duration and is calculated as follows: *hIa*: hI,norm/academic age, (where: academic age: number of years elapsed since first publication).

Politicians involved in this delicate task (such as ministry of the University) should seriously put hand to a vigorous review of the rules governing the progression in academic careers after a necessary consultation with experts in the field. The critical points raised in this paper could represent a useful base to reconsider the rules for the NSH.

## Data Availability

Not applicable.
